# Auditory Cortical Detection and Discrimination Correlates with Communicative Significance

**DOI:** 10.1371/journal.pbio.0050173

**Published:** 2007-06-12

**Authors:** Robert C Liu, Christoph E Schreiner

**Affiliations:** 1 Department of Biology, Emory University, Atlanta, Georgia, United States of America; 2 Center for Behavioral Neuroscience, Atlanta, Georgia, United States of America; 3 W. M. Keck Center for Integrative Neuroscience, University of California at San Francisco, San Francisco, California, United States of America; 4 Department of Otolaryngology–Head and Neck Surgery, University of California at San Francisco, San Francisco, California, United States of America; Hebrew University, Israel

## Abstract

Plasticity studies suggest that behavioral relevance can change the cortical processing of trained or conditioned sensory stimuli. However, whether this occurs in the context of natural communication, where stimulus significance is acquired through social interaction, has not been well investigated, perhaps because neural responses to species-specific vocalizations can be difficult to interpret within a systematic framework. The ultrasonic communication system between isolated mouse pups and adult females that either do or do not recognize the calls' significance provides an opportunity to explore this issue. We applied an information-based analysis to multi- and single unit data collected from anesthetized mothers and pup-naïve females to quantify how the communicative significance of pup calls affects their encoding in the auditory cortex. The timing and magnitude of information that cortical responses convey (at a 2-ms resolution) for pup call detection and discrimination was significantly improved in mothers compared to naïve females, most likely because of changes in call frequency encoding. This was not the case for a non-natural sound ensemble outside the mouse vocalization repertoire. The results demonstrate that a sensory cortical change in the timing code for communication sounds is correlated with the vocalizations' behavioral relevance, potentially enhancing functional processing by improving its signal to noise ratio.

## Author Summary

Like a student in a foreign country immersed in an unfamiliar language or a young mother trying to decipher her baby's cries, we all encounter initially meaningless sounds that in fact carry meaning. As these sounds gain significance, we become better at detecting and discriminating between them. How does this occur? What happens in our brain to facilitate this improvement? We explored these questions in a mouse model by measuring how neurons in the auditory cortex of female mice respond when the ultrasonic calls of mouse pups are played back to the animals. Earlier studies demonstrated that mothers, but not virgin females, recognize these calls as behaviorally significant. Our results indicate that the timing and magnitude of the auditory cortical responses to these communicative sounds differ between these two groups of female mice and that this difference may provide the auditory system in mothers with the capacity for detecting and discriminating pup calls. The results demonstrate that behavioral significance can be correlated with quantifiable functional improvements in the sensory cortical representation of a communication sound.

## Abstract 3

Pup calls produce quicker and larger neural responses, which convey more information for pup call detection and discrimination, in the auditory cortex of mother mice compared with virgin female mice.

## Introduction

A central question in neuroscience is how behaviorally relevant sensory signals are encoded by the brain. In the context of species-specific communication, this issue is complicated by the fact that many sounds with the same meaning are variable in their physical characteristics, such as speech phonemes spoken by different people [1,2]. In some cases, only the message itself is relevant, and just its detection over background noise is necessary; in other cases this variability discriminates between various speakers. What aspects of the neural code carry information for the detection and discrimination of such naturally varying sounds, and does their behavioral relevance affect their encoding?

These questions have not been fully addressed in mammals, despite a rich literature on the neural representation of communication sounds, particularly in auditory cortex. Most research has focused on the selectivity of cortical neurons for intraspecies communication calls of primates [3–7], guinea pigs [8,9], bats [10,11], and cats [12]. Neurons have generally not been found to be call specific in their response [13]. This poor selectivity does not imply an absence of information that could be useful for detecting and discriminating calls: some neurons may be more informative than others, even if they are not call selective. Evaluating this possibility first requires a quantitative characterization of vocalization variability, as has been done for the marmoset [14] and bat [15]. In the latter, this has led to the conclusion that the average temporal pattern of neural responses helps discriminate categories of calls with very different acoustic structure [16]. These animal models do not reveal though whether the significance of the communication sound per se impacts neural coding, as some cross-species studies suggest [13,17]. Therefore, a model system is needed in which the encoding of variable vocalizations can be quantitatively compared between animals (of the same species) for which specific sounds either do or do not carry communicative significance.

The mouse ultrasound communication system provides such an opportunity [18–20]. The emission of ultrasonic calls by isolated mouse pups acts as a communication signal to elicit a search and retrieval by mouse mothers [20,21]. The variability in the acoustic parameters of these calls has been extensively characterized [19], laying the foundation for quantitative neural-coding studies. In two-alternative choice tests, mothers preferentially approach pup-like ultrasounds over a neutral sound not in the mouse vocal repertoire and can even discriminate ultrasounds based on frequency, duration, and bandwidth [22–24]. This preference is a clear indication that pup calls carry communicative significance for mothers, a significance that is not recognized by pup-naïve virgins, which do not favor these ultrasounds [25]. This contrast therefore supplies a natural control animal group for investigating whether and which aspects of the neural code correlate with communicative significance.

We pursued this by recording auditory cortical spiking activity in response to natural mouse pup isolation calls from anesthetized mothers (whose pups were weaned within one week prior to experiments) and virgins (which were never housed as adults with pups). The auditory cortex was chosen since immediate early gene expression (c-Fos) [26] and neuronal responsiveness to the call bout structure [27] hint that this area reflects the recognition of pup calls by mothers. Here, we introduce a novel methodology to evaluate the information that auditory cortical neurons carry for the detection and discrimination of pup calls and test whether differences in information encoding exist between mothers and virgins. Because behavioral preference is a consequence of sensory, motivational, decisional, and motor processing, it is not immediately obvious that the neural firing in a sensory cortical area will be correlated with communicative significance. We found that the timing of the information about pup calls in cortical responses of mothers is significantly different from pup-naïve virgins, resulting in improved detection and discrimination ability for behaviorally relevant communication sounds.

## Results

### Neural Response to Pup Calls

**Figure 1:**
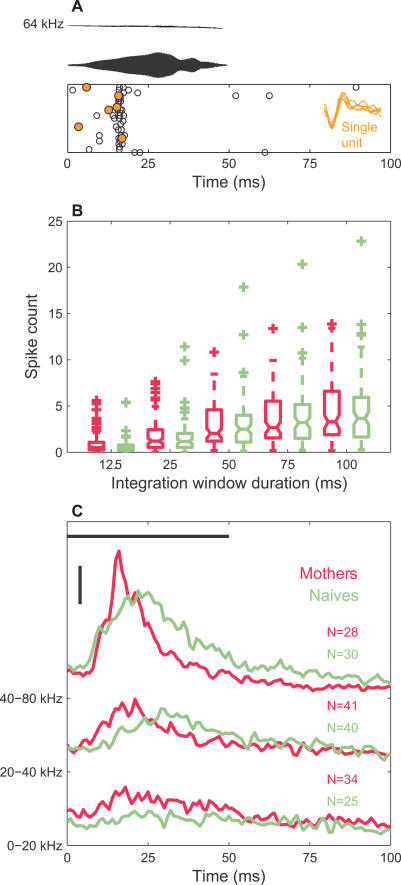
Responses from Mothers and Naïve Females to a Typical Mouse Pup Call (A) Frequency trajectory (top) and amplitude waveform (middle) of the pup call (64-kHz median frequency, 50-ms duration, 65 dBSPL) are shown. At the bottom is the raster plot of responses from a MU (white circle) and extracted SU (gold circle) to 24 presentations of the call. (B) A whisker plot is shown of the average number of spikes (across 24 trials) in progressively larger integration windows, triggered 6 ms after the onset of a call. The notched lines indicate medians; the ends of each box mark the upper and lower quartiles; the whiskers denote the most extreme data values within 1.5× the interquartile range; and the crosses mark the outliers. No significant differences (*p* > 0.05) between mothers (magenta, *n* = 86) and naïve females (sage, *n* = 74) were found in the median (two-sided rank sum test), mean (two-sample t-test), or distribution (two-sample Kolmogorov-Smirnov [KS] test) for spike counts at any window duration. The same color designation for mothers and naïve females is used in all plots. (C) PSTH (smoothed at 2 ms) is shown segregated by recording sites' CF into three ranges (0–20 kHz, 20–40 kHz, and 40–80 kHz). The black, horizontal bar indicates the playback period. The vertical scale bar equals 50 spikes/s. Fitting the populations' PSTHs to the empirical function, *a*(*t* − *t*_0_)^3^ exp(−*b*(*t* − *t*_0_)) + *c*, the peak times and widths could be extracted (fit with MATLAB cftool, restricted to the time interval from six to 71 ms relative to the stimulus onset, resulting in adjusted *R*-square values between 0.87 and 0.96). The highest CF group showed an earlier (16.6 ms versus 21.2 ms) and narrower (15.1 ms versus 27.4 ms half max) peak in mothers compared to naïve females, respectively. This was also true of the less strongly driven middle CF group (18.2 ms versus 29.5 ms peak and 20.1 ms versus 28.1 ms width).

A typical pup call evoked a strong, time-locked neural response in the auditory cortex of anesthetized mice (Figure 1A). Similar average spike counts were elicited from both mothers and pup-naïve virgin female mice (Figure 1B). This might lead to the conclusion that auditory cortical processing is not sensitive to the behavioral significance of a communication sound. However, looking closely at the time course of the responses (Figure 1C), important differences between animal groups appeared. The peristimulus time histogram (PSTH) generally peaked earlier in mothers, with a larger and narrower response. This was most prevalent for recording sites having characteristic frequencies (CFs) (frequency of the lowest amplitude tone that elicits a response) near the pup call frequency range (40–80 kHz), but was also seen for mid-CF sites (20–40 kHz). Thus, might the timing of neural responses carry information about behavioral relevance?

**Figure 2:**
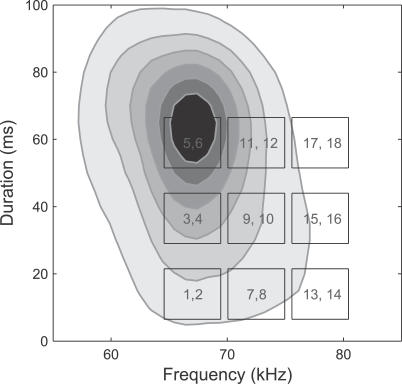
Collection of Natural Pup Calls The contour plot shows the probability that a natural pup calls has a specific combination of frequency and duration, with darker gray corresponding to higher probability (contours at probability densities of 0.19, 0.37, 0.56, 0.75, and 0.93/s/kHz). Only the main pup call cluster near 67 kHz and 59 ms is shown [19]. A grid of nine frequency-duration regions (centered at 67, 72.5 and 78 kHz and 14, 36.5 and 59 ms) laid across both high and low probability calls was used to condition the selection of playback calls on the basis of frequency and duration. Two natural calls from each region were randomly selected.

To investigate this, we focused on two behaviorally important functions in communication: detection (“did a call occur?”) and discrimination (“is one call different from another?”). In practice, these tasks are complicated by the natural variability of communication calls. For example, both the median frequency and duration vary due to individual pup differences as well as age-related changes [19]. To test how neural responses encode this natural variation, we played back 18 different pup calls, two each chosen randomly from nine regions in the frequency-duration plane (Figure 2). This collection included both high and low probability calls that varied systematically in these two parameters (rather than only higher probability calls that would have been chosen by a purely random selection strategy).

**Figure 3:**
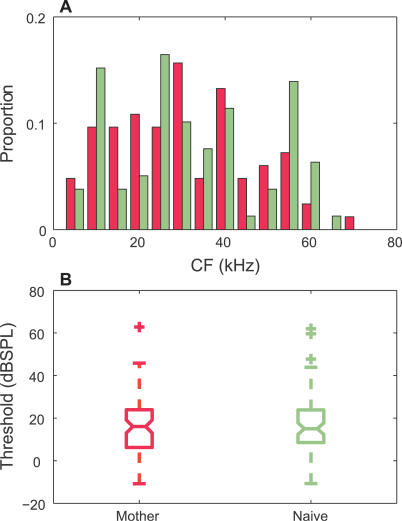
Comparison of Tone Responses from MU Recordings Sites Contributing to the Information Analysis (A) Histograms show CFs (5-kHz bins) for sites in mothers (*n* = 83 out of 96 sites had identifiable CFs) and naïve females (*n* = 79 out of 102 sites). No significant differences were found between the distributions (two-sample KS test, n.s.). (B) A whisker plot of the tone thresholds is shown. The notched lines indicate medians; the ends of each box mark the upper and lower quartiles; the whiskers denote the most extreme data values within 1.5× the interquartile range; and the crosses mark the outliers. No significant differences were found between the distributions (two-sample KS test, n.s.).

**Figure 4:**
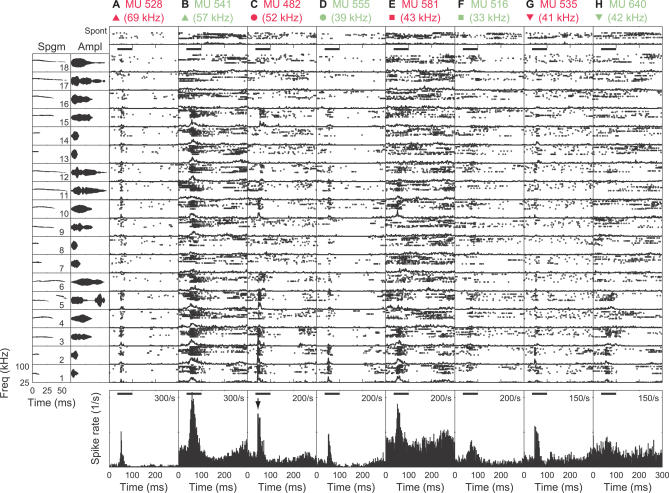
Example MU Responses to Natural Pup Calls (Left column) Frequency trajectories and amplitude waveforms of the 18 pup calls are presented. All calls were simple whistles with different frequency and amplitude modulations. They increased systematically in duration and frequency. (A–H) a raster plot of MU responses is shown. Each subpanel includes both the raster of individual spike responses from 12 trials, as well as its corresponding PSTH (2-ms bins). The top panel shows the spontaneous activity. The middle 18 panels show the responses to individual pup calls presented at the start of the horizontal black bar, and continuing for the duration of each call (bar's length indicates the duration of the longest stimulus, 65 ms). The larger, bottom panel shows the overall PSTH to all the pup calls, with a spike rate scale along the right edge of each panel. The labeling at the top of each column indicates the recording site (magenta for mothers and sage for naïve females) and its CF (in parentheses). The symbols are used for identifying each of the sites in subsequent figures. The downward arrow in the overall PSTH plot for panel (C) indicates the time bin used for the illustrations in Figure 6.

We collected multiunit (MU) spike activity in response to these calls at a population of recording sites across the mouse auditory cortex of mothers and naïve females (see Materials and Methods). The tonal CFs and thresholds of these sites were not significantly different between the two animal groups (*p* > 0.05) (Figure 3). The 18 calls (Figure 4, left column) elicited a variety of different responses. Figure 4A, 4C, 4E, and 4G (4B, 4D, 4F, and 4H) show the raster of spike activity from four MU sites in mothers (naïve females), along with their respective spontaneous activities (top panels). These examples were selected to convey the range of strong and weak responses observed in both animal groups. The overall firing rate elicited by all calls (bottom panels) rose sharply just after sound onset for many sites. MU 482 responded selectively to some vocalizations (such as numbers 1–3) and not others (like numbers 7–9), with slight shifts in latency (compare numbers 10 and 15). MU 528 responded only at the onset of nearly all the calls, albeit with different firing probabilities for different calls.

**Figure 5:**
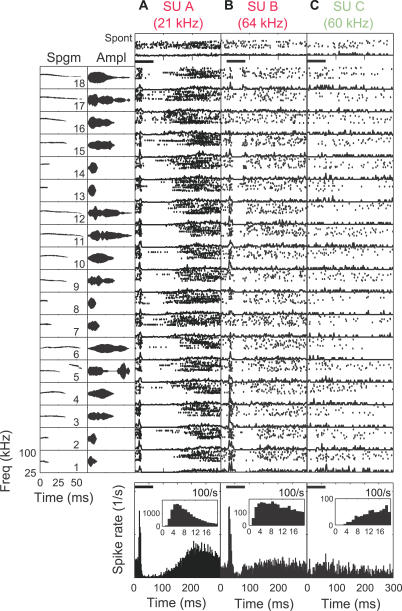
Example SU Responses to Natural Pup Calls (Left column) See Figure 4 legend. (A–C) See Figure 4 legend, except for well-isolated SUs recorded from mothers. The interspike interval distribution (1-ms bins) is shown as an inset in the overall PSTH panels. The lack of short interspike intervals within the absolute refractory period (1 ms) supports the SU designation. The number of trials collected depended on how long each SU was maintained: 50 trials for SU A, 24 for SU B, and 14 for SU C.

Two well-isolated single units (SUs) from mothers (SU A and SU B in Figure 5A and 5B, respectively) showed similar response features: SU A was an onset responder to all calls, while SU B responded in a slightly more graded fashion to different calls. SU C from a naïve female responded weakly to pure tones (unpublished data), but had an identifiable CF near 60 kHz. Its overall firing to pup calls, however, showed only a slight elevation during the calls relative to its spontaneous firing.

### Call Detection Information

To make quantitative statements about the processing of these communication sounds, we evaluated the information that neural responses conveyed for call detection and discrimination. In general, information about a stimulus *s* may be provided by the entire time course of a response *r*. We analyzed this response time course in 2-ms bins, treating each bin as independent. In principle, a pattern of spikes may convey more or less information than the contribution from each spike [28,29]. We did not integrate the information over time, so our approach ignored potential synergy and redundancy for spikes lying in different 2-ms bins. However, we could nevertheless reveal coding differences that were correlated with communicative significance, the main objective of this work.

Intuitively, information about a stimulus is gained from a response if the latter reduces the uncertainty about what stimulus occurred. For detection, different calls within a category are equivalent, and the acoustic variability is immaterial. The uncertainty is only about whether any call occurred relative to silence. Thus, an ideal detector would generate the same spike response regardless of the call—a response different from its spontaneous firing. Formally, the mutual information between the stimulus possibilities (*s* = “call” or “no call”) and response possibilities (spike count in a 2-ms bin) quantifies how much the latter changes our uncertainty in the former (see Materials and Methods).

**Figure 6:**
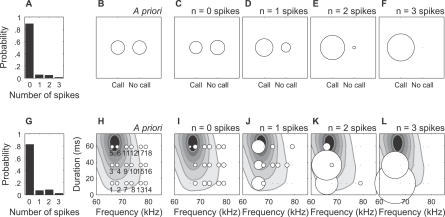
Detection and Discrimination Information from Spike Counts in a 2-ms Bin, 10 ms after Stimulus Onset for MU 482 (A) The overall probability that a specific number of spikes was observed during either a “call” or “no call” (spontaneous activity) is shown. (B) Initial probability for a “call” or “no call” is shown. The diameter of each circle is proportional to the probability. Both stimulus possibilities were considered equally likely before a response was observed. (C) The conditional probability for a “call” or “no call” given that no spikes were observed is presented. Zero spikes did not substantially improve the uncertainty. (D–F) Conditional probability for a “call” or “no call” given that one, two, or three spikes was observed, respectively. With each larger spike count, the probability for “call” increased, thus reducing the uncertainty in the stimulus and contributing detection information, weighted by the probability for that spike response (A). (G) The overall probability that a specific number of spikes was observed during any of the 18 calls is shown. (H) The initial probability for each call is presented. The diameter of each circle is proportional to the probability. All calls were considered equally likely before observing the response. (I) Conditional probability for each call given that no spikes were observed. Zero spikes did not substantially improve the uncertainty. (J–L) The conditional probability for each call is shown given that 1, 2 or 3 spikes were observed, respectively. With each larger spike count, the probability increased that one of the lower frequency calls occurred, thus narrowing the uncertainty in the stimulus, and contributing discrimination information, weighted by the probability for that spike response (G).

Figure 6A–6F illustrates this for MU 482 at a time bin *t* corresponding to the peak in the PSTH (arrow in the bottom panel of Figure 4C). We grouped all 18 calls together into a single category, thereby ignoring the identity of individual vocalizations. Before observing the response *r* at *t*, both the “call” and “no call” possibilities were considered equally likely (log_2_[2] = 1 bit of uncertainty), so that their probabilities (diameter of the circular icons in Figure 6B–6F) were the same. If no spikes were observed, the two stimulus possibilities were still about the same (Figure 6C). However, if one, two, or three spikes were observed, it was progressively more likely that a pup call occurred (Figure 6D–6F). Thus, the detection uncertainty was reduced by observing *r*, and information was gained. The total amount of information contributed by this time bin was 0.1 bits, defined as the average change in uncertainty from each response possibility (zero, one, two, or three spikes), weighted by the probability of that response (Figure 6A). This analysis therefore provided a quantitative measurement of the ability of this neural response to convey information for detecting the behaviorally important communication call.

### Call Discrimination Information

A similar analysis can be applied to quantify how well the neural response discriminates calls. In this case, differences between pup isolation calls are important, and the uncertainty is about which of the 18 calls occurred. An ideal discriminator would fire in a unique manner for each of the *s* = “call 1” to *s* = “call 18” calls.

How much information do real auditory cortical responses provide for discriminating calls? Figure 6G–6L illustrates this assessment for MU 482, at the same time bin considered for detection, above. A priori, all calls were considered equally likely, resulting in log_2_[18] = 4.2 bits of uncertainty (Figure 6H). Conditioning on the different possible spike count responses (Figure 6G), some stimuli clearly became more likely. It was found that zero spikes did not markedly change the uncertainty (Figure 6I). However, if one spike was observed, one of the lower frequency calls most likely elicited that response (Figure 6J). The stimulus uncertainty was further reduced by two or three spikes (Figure 6K–6L), since only five or two of the 18 calls, respectively, were likely. Overall, this time bin provided 0.6 bits of discrimination information for MU A, thereby quantifying the neural ability to tell these different communication calls apart.

### Information Time Course

**Figure 7:**
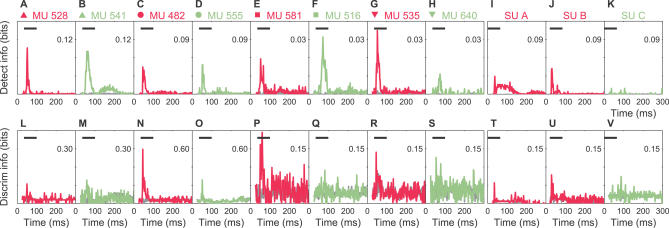
Time Course of the Detection and Discrimination Information for Examples in Figures 4 and 5 (A–H) Detection information for MUs is shown. Each panel corresponds to a site in Figures 4 or 5, as indicated by the labels and symbols. Most sites for both mothers (magenta) and naïve females (sage) exhibited peaks in detection information above the randomized control estimate (gray) during the stimulus presentation period. Note the change in scale (indicated to the right) between different panels. (I–K) Detection information for SUs is shown. SU A from a mother was notable in that information remained significantly above the randomized control throughout and even after the duration of the stimuli. This corresponded to the period when calls inhibited neural firing relative to the spontaneous background, showing that zero spikes can be informative. Scale is to the right in each panel. (L–S) Discrimination information for MUs for the same site in each column is presented. Some sites with good detection information (e.g., MUs 533 and 541) showed only weak discrimination information. Note the change in scale (indicated to the right) between different panels. (T–V) Discrimination information for SUs is shown.

Using this methodology, we derived time courses for the information each independent time bin in the response conveyed for detection (Figure 7A–7K) and discrimination (Figure 7L–7V), for the examples in Figures 4 and 5. For comparison, we also randomized trials across the stimulus possibilities (see Materials and Methods) so that no information was in principle available. Since the noise in the spike counts from finite trials can cause both a bias and fluctuations in the information estimates [30,31], the randomized estimate provided a baseline for comparing whether peaks in the information were significant.

MU 482 exhibited large peaks above the randomized estimate (gray lines) in both detection and discrimination information soon after the stimulus onset, as expected from its consistent yet systematically varying responses to different calls. On the other hand, MU 528 showed appreciable detection but very little discrimination information, as expected from the onset nature of its responses. SU A exhibited an extended period of detection information lasting beyond the duration of the call (Figure 7I). This matched the interval when calls suppressed spiking relative to the spontaneous activity (Figure 4C) and demonstrates that the absence of spikes can also be informative. Furthermore, the naïve female example, SU C (Figure 7K and 7V), produced time courses that were fairly similar between the actual information and the randomized control information. This was not surprising, given the unit's poor response to individual calls (Figure 5C). These examples demonstrate how our information analysis quantitatively summarized the complex neural coding of natural calls, yielding results that were consistent with our qualitative impressions.

### Information Comparison between Mothers and Naïve Females

**Figure 8:**
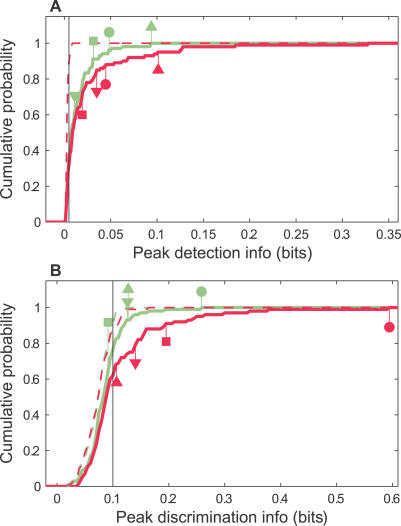
Cumulative Probability Distributions for Peak Information Values across the MU Population (A) The lines plot the cumulative probability that a site had a peak detection information value equal to or less than the abscissa. (B) The lines plot the cumulative probability that a site had a peak discrimination information value equal to or less than the abscissa. The peak (detection or discrimination) information was determined for each MU site as the maximum information within either 5–70 ms after the onset of the calls (solid lines), or within 365–430 ms after the onset (dashed lines). The former represents the peak information during the response to the calls. The latter provides an estimate of the noise in our information calculations since little information was expected during this late period, when the activity had essentially returned to the spontaneous level. The symbols identify the peak information values in response to calls of corresponding sites in Figure 4. During the interval in response to calls, the distribution of peak information values in mothers clearly differed from naïve females, unlike the case for the late period. This difference appeared at higher peak information values, rather than across all information values. One reason the cumulative distributions coincided at lower information values may be due to noise or bias in our estimation procedure. This would have been more likely for the discrimination case (B), where the cumulative distribution for the late period estimate tracked that of the call period up to ~0.1 bits. This value corresponds to the naively expected bias for 18 stimulus possibilities and four response possibilities [31]. However, realistic simulations (see Materials and Methods) indicate that our estimation procedure is accurate when the true information is higher than ~0.1 bit. Indeed, if our estimation procedure were dominated by a systematic bias, the distribution of information values for the two animal groups would be quite similar across the whole range of values, as was the case for the late period estimate (dashed lines). An alternative explanation for the coincidence of the cumulative distributions at lower values might be that the information improvement between naïve females and mothers only involves sites that already convey some minimum amount of information about the calls. This seems plausible for detection since its systematic bias was negligible. Nevertheless, to avoid possibly contaminating our comparisons between the two animal groups, we set a significance threshold (thin vertical black lines in each panel) for peak information values of 0.005 bits (detection) and 0.1 bits (discrimination). Sites with peak information estimates above these were considered valid. These thresholds corresponded to 90% of the largest peak detection and discrimination information values measured during the late period.

Next, the MU neural population was analyzed as described above to see whether mothers and naïve females coded pup calls differently. Since we were not very restrictive in selecting recording sites (see Materials and Methods), many showed rather poor information; with peak information values (time of maximum detection information) during the calls that varied little from peak values long after the calls were presented (i.e., after the spike rate had returned to the spontaneous level). To avoid claiming that such sites carry significant information, we assessed the distribution of peak information during a very late period in the activity (arbitrarily chosen at 365 to 430 ms after call onsets). The cumulative probability distributions of this peak information are shown by the dashed lines in Figure 8A and 8B for detection and discrimination, respectively. As expected, there was virtually no difference between mothers (magenta) and naïve females (sage). This was a period when information about the calls should be minimal, and any residual information is likely dominated by noise or bias in our estimation procedure, which would be common to both animal groups. A further check of the cumulative distributions of the peaks in the randomized control information also showed no difference between mothers and naïve females (unpublished data), providing further confidence that our estimation procedure did not artificially inflate the information values of one animal group over the other.

In contrast, when the peak information during the response to the calls was compared between sites for mothers and naïve females (peak between 5–70 ms after call onset, an interval equal to the longest call duration) (solid magenta and sage lines in Figure 8, respectively), a clear difference was apparent. The cumulative probability distributions showed a sizeable gap between the two animal groups, with the mothers exhibiting a larger proportion of sites with higher detection and discrimination information (see Figure 8 legend for further details). This was the first indication that the coding of pup calls differs between animals with and without exposure to and experience with pups.

**Figure 9:**
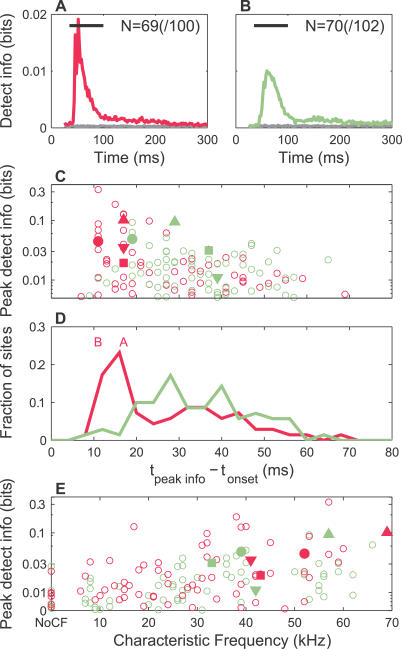
Population Summary of Detection Information (A) The average time course of detection information in mothers, for significant recording sites (see text and Figure 8 legend) is presented. Both the population-averaged actual (magenta) and randomized (gray) information are shown. (B) The average time course of detection information in naïve females, for all recording sites is presented. Both the actual (sage) and randomized (gray) information are shown. By fitting the average information time courses to the empirical function, *a*(*t* − *t*_0_)^3^ exp(−*b*(*t* − *t*_0_)) + *c* (fits had adjusted *R*-squares of 0.83 for mothers and 0.98 for naïve females), the peak in the population-averaged information time trace was found to be 1.3× larger in mothers than in naïve females (if actual peaks relative to the randomized baselines are used instead, mothers were 1.9× larger than naïve females). (C) Peak detection information versus latency for each significant MU site is presented. The y-axis is plotted on a logarithmic scale to clearly separate individual points. The symbols identify results for the corresponding sites in Figure 4. Even at the level of individual recording sites, there was a clear tendency for high information sites in mothers to have shorter latencies. This was less so for naïve females. (D) Histogram of peak detection information times (4-ms bins, relative to stimulus onset), for sites with significant information is presented. The distribution in mothers (magenta) was shifted to shorter latencies compared to naïve females (sage). The two distributions were significantly different (*p* ≪ 0.05, two-sample KS test), as were their medians (*p* ≪ 0.05, two-sided rank sum test) and means (*p* ≪ 0.05, two-sample t-test). The peak information times for SU A and SU B are indicated at the top of the panels. They coincided with the main peak in the distribution for mothers. (E) Peak detection information versus CF for each significant MU site is presented. The y-axis is plotted on a logarithmic scale to clearly separate individual points. The symbols identify results for the corresponding sites in Figure 4. There was a significant correlation between peak detection information and CF for both mothers (*r* = 0.35, 95% confidence interval 0.11–0.55, *p* ≪ 0.05) and naïve females (*r* = 0.45, 95% confidence interval 0.23–0.63, *p* ≪ 0.05).

**Figure 10:**
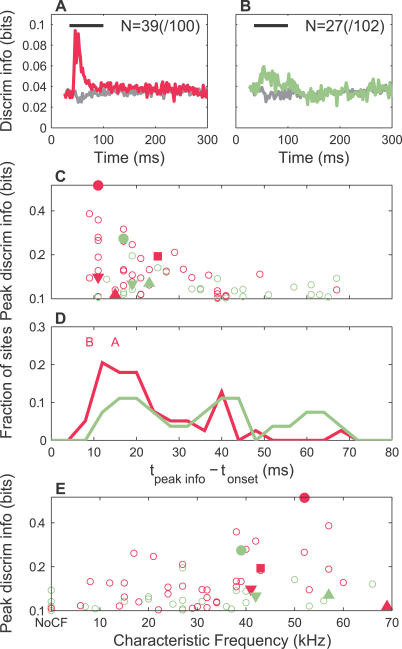
Population Summary of Discrimination Information (A) Average time course of discrimination information in mothers for significant recording sites is presented (see text and Figure 8 legend). Both the population-averaged actual (magenta) and randomized (gray) information are shown. (B) Average time course of discrimination information in naïve females for all recording sites is presented. Both the actual (sage) and randomized (gray) information are shown. By fitting the average information time courses to the empirical function *a*(*t* − *t*_0_)^3^ exp(−*b*(*t* − *t*_0_)) + *c* (fits had adjusted *R*-squares of 0.84 for mothers and 0.42 for naïve females), the peak in the population-averaged information time trace was found to be 3.4× larger in mothers than in naïve females (if actual peaks relative to the randomized baselines are used instead, mothers were 2.3× larger than naïve females). (C) Peak discrimination information versus latency for each significant MU site is presented. The y-axis is plotted on a logarithmic scale to clearly separate individual points. The symbols identify results for the corresponding sites in Figure 4. Even at the level of individual recording sites, there was a clear tendency for high information sites to have shorter latencies, which was most apparent for mothers. (D) Histogram of peak discrimination information times (4-ms bins, relative to stimulus onset) for sites with significant information is presented. The distribution in mothers (magenta) was shifted to shorter latencies compared to naïve females (sage). The two distributions were significantly different (*p* < 0.05, two-sample KS test), as were their medians (*p* ≪ 0.05, two-sided rank sum test), and means (*p* ≪ 0.05, two-sample t-test). The peak information times for SU A and SU B are indicated at the top of the panels. They coincided with the main peak in the distribution for mothers. (E) Peak discrimination information versus CF for each significant MU site is presented. The y-axis is plotted on a logarithmic scale to clearly separate individual points. The symbols identify results for the corresponding sites in Figure 4. There was a correlation between peak discrimination information and CF for mothers (*r* = 0.36, 95% confidence interval 0.04–0.61, *p* < 0.05), but not for naïve females (n.s.). This correlation was weaker than the case of detection, perhaps reflecting the tendency that even lower CF sites (e.g., around 20 kHz) could have high peak discrimination information (~0.2 bits).

This was further evident in a comparison of the average (over sites) time course for detection and discrimination information (Figures 9 and 10). Restricting ourselves to only those sites that likely carried significant peak information (to the right of the black threshold line in Figure 8, see Materials and Methods), the neural responses in mothers (Figure 9A) conveyed more detection information on average than naïve females (Figure 9B). Even when all sites were included, these conclusions were the same. In particular, mothers showed a strong, early peak that was lacking in naïve females (empirically fit peak at 18 ms versus 29 ms, relative to the stimulus onset, respectively; see Figure 9 legend). This can also be seen by comparing the peaks of individual sites, as illustrated in Figure 9C. Plotted on a logarithmic scale for clarity, higher information sites had shorter latencies, especially for mothers. Indeed, the distribution of peak information latencies was shifted to shorter times (max at 16 versus 28 ms) in mothers compared to naïve females (Figure 9D). These results suggest that neurons in mothers provide earlier and greater information for detecting pup calls.

Discrimination improvement was even more striking. When all sites were considered, the average information time course peaked strongly for mothers, but barely changed from the randomized control for naïve females. When only the most significant sites were averaged together and the resulting peaks were numerically fit, mothers had a relative information peak around three times larger, and earlier (14.6 ms versus 21 ms), than naïve females (Figure 10A versus 10B, see legend). The sites that contributed the greatest peak discrimination information were again clustered at the shortest latencies, particularly for mothers (Figure 10C). The distribution of these peak information latencies was broad for both groups, but weighted toward shorter latencies in mothers (Figure10D). Finally, there were significantly more sites in mothers that conveyed discrimination information beyond our threshold (*p* < 0.05, test of proportions). Taken together with the detection results, our study suggests a correlation between the communicative significance of a sound category to an animal and that animal's auditory cortical detection and discrimination processing of those sounds.

### Frequency and Duration Information

To try to understand the origin of this improved information in mothers, we considered several additional analyses. Since the calls varied systematically in frequency and duration, we asked whether the responses discriminated between calls because they provided information specifically about these acoustic parameters. We reanalyzed the information by grouping calls first according to the three different frequency ranges from which they were selected (frequency information) and also according to the three different duration ranges (duration information). This ignored all other acoustic differences between calls, such as amplitude envelope variations, and only considered how the responses informed about the consistent acoustic parameter—frequency or duration.

**Figure 11:**
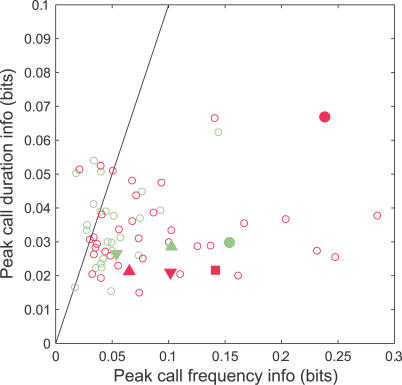
Frequency and Duration Information For each site in mothers and naïve females that conveyed significant call discrimination information, the time courses of information about call frequency (three ranges) and call duration (three ranges) were also computed. The maximum (over time) information for both frequency and duration at each site were then determined and plotted. Sites generally conveyed more information about the call frequency rather than the duration (points mostly on the right of the diagonal). Furthermore, the most informative sites in mothers clearly conveyed more frequency information than those from naïve females. Thus, the improvement in call discrimination probably arose from changes in the spectral encoding of these natural calls. The symbols identify results for the corresponding sites in Figure 4.

Figure 11 plots the maximum (over time) information available for distinguishing call frequency and duration (see Materials and Methods). Only those sites with significant call discrimination information are shown. For both mothers and naïve females, there was a tendency towards better frequency rather than duration information (data lie mainly to the right of the diagonal). Furthermore, more sites in mothers had large frequency information, suggesting that the mothers' improved information for call discrimination may be related to a neural change in frequency sensitivity over this very narrow range of pup call frequencies. Indeed, for both animal groups, discrimination information was higher at sites with greater frequency information. Using the MATLAB analysis of covariance tool AOCTOOL, the linear regression slope of 0.98 was significantly different from 0, *p* ≪ 0.05, and the slopes for mothers and naïve females were not significantly different from each other, *F*(1,62) = 0.8, not significant (n.s.).

Importantly, although frequency sensitivity appears to be an important factor in improving the discrimination information, the improvement was not restricted simply to units with higher CFs, which might be expected to have better frequency sensitivity in the pup call range. In fact, discrimination information was generally better in mothers across all CFs. After taking into account the CF dependence of the discrimination information, which was fitted at 0.0016 bits/kHz (an analysis of covariance showed that the slopes were not significantly different between mothers and naïve females, *F*[1,57] = 1.67, n.s.), a significant main effect due to animal group was found (*F*[1,58] = 6.53, *p* < 0.05).

### Noncommunicative Sound Ensemble

We next wondered whether the coding difference between mothers and naïve females was specific to pup isolation calls, or whether it might generalize to a noncommunicative sound ensemble as well. Since the recognition of an ultrasound signal as a pup call by a mother can depend on spectral cues [22,24,32], we frequency halved the natural pup call frequencies (see Materials and Methods) to generate a collection of behaviorally irrelevant but acoustically related sounds. Because of the logarithmic frequency scale of the basilar membrane, this ensemble spanned an extent along the cochlea comparable to that of the original calls. We presented the sounds at a random subset of recording sites and computed the detection and discrimination information for these translated calls.

**Figure 12:**
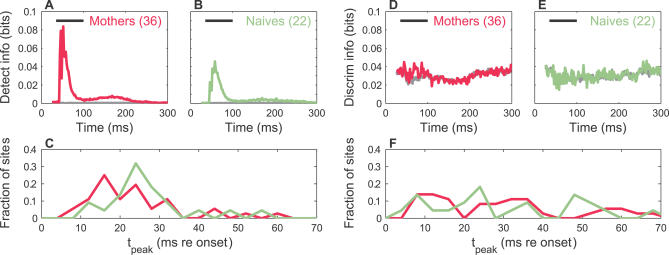
Detection and Discrimination Information for a Noncommunicative Sound Ensemble (Frequency-Halved Pup Calls) (A) Average time course of detection information in mothers for all sites where this ensemble was presented. Both the measured (magenta) and randomized (gray) information are shown. (B) Average time course of detection information in naïve females for all sites where this ensemble was presented. Both the measured (sage) and randomized (gray) information are shown. The information peak (above the mean randomized information) for mothers was 1.5× larger than that for naïve females, irrespective of whether all sites or only those with information z-scores exceeding *z*_c_ were used. (C) Histogram of peak detection information times (4-ms bins, relative to stimulus onset) for all sites is presented. The distribution for mothers was not significantly different from that of naïve females (two-sample KS test, n.s.), neither were their medians (two-sided rank sum test, n.s.), nor their means (two-sample t-test, n.s.). (D) Average time course of discrimination information in mothers for sites with significant information is presented. Both the measured (magenta) and randomized (gray) information are shown. (E) Average time course of discrimination information in naïve females for sites with significant information is presented. Both the measured (sage) and randomized (gray) information are shown. (F) Histogram of peak discrimination information times (4-ms bins, relative to stimulus onset), for all sites. The distribution for mothers was not significantly different from that of naïve females (two-sample KS test, n.s.), neither were their medians (two-sided rank sum test, n.s.), nor their means (two-sample t-test, n.s.).

This ensemble excited the recording sites quite well since their ~35 kHz frequency was closer to the frequency of the minimum behavioral hearing thresholds for mice [33]. Therefore, it was not surprising to see that both mothers and naïve females showed strong detection information (Figure 12A and 12B). However, the distribution of latencies to the peak detection information was not significantly different between the two groups (Figure 12C). Therefore, although there may be some generalized improvement in sound detection information in mothers, it occurred without significantly changing the timing of the information. Furthermore, unlike the natural pup call case, there was virtually no information on average for discriminating these frequency-divided sounds in either mothers or naïve females (Figure 12D and 12E). Peak information latencies were also not significantly different (Figure 12F). Hence, the change from the naïve to maternal state did not appear to substantially affect the neural discrimination information for these noncommunicative signals. This is consistent with the idea that behavioral relevance is an important factor for altering the auditory cortical coding of a sound ensemble.

## Discussion

Our main new finding was that the behavioral relevance of an intraspecies communication call is correlated with changes in the timing of the auditory cortical spiking response. Specifically, our analysis revealed that the information neural responses convey for detecting and discriminating natural vocalizations reaches a larger and earlier peak in animals for which the calls have communicative significance. Moreover, the data suggest that sites conveying the most information do so with the shortest latencies, a property that may improve the synchronization of relevant neurons, as well as the signal to noise level at the input of downstream areas. Finally, better frequency encoding of calls, regardless of CF, appears to be primarily responsible for improving call discrimination information in the auditory cortical responses of mothers.

These results provide evidence in a novel mammalian model that the timing of spikes, and not just the average spike count, is an important aspect of the neural code for communication sounds. Two caveats should be mentioned. First, this study was performed in anesthetized animals, where stimulus control and animal state can be straightforwardly controlled. Ultimately though, the coding of behaviorally relevant stimuli should be tested in nonanesthetized preparations as well. Second, our conclusions are primarily based on MU data, although they were apparently not very sensitive to the number of contributing neurons (see Materials and Methods); and our SU examples agreed with our findings.

The idea that spike timing might be important for behaviorally relevant vocalization encoding within the auditory system has been implied in earlier work. A pioneering study in marmosets found that the auditory cortical discharge to a species-specific twitter call was much more synchronized across recording sites than would be predicted by the spectrographic representation of the sound, with many neurons firing earlier than expected [5]. Furthermore, a recent study in the zebra finch homologue of the inferior colliculus reported that neurons fired earlier, more precisely, and synchronously to natural bird songs than to behaviorally irrelevant modulation-limited noise [34]. Another study in the zebra finch homologue of auditory cortex found that finer (10 ms) rather than coarser temporal resolutions were optimal for spike trains to discriminate different bird songs [35]. These studies looked only at animals for which the natural vocalizations were already behaviorally relevant; the results could therefore be due to evolutionary, developmental, and/or experience-dependent mechanisms [36].

To our knowledge, the current work demonstrates for the first time that the neural code for communication sounds in adult animals can change (because of either experience or possibly hormonal mechanisms) as the significance is acquired, and that this plasticity can quantitatively improve information processing for specific communicative functions. This goes beyond a parallel study in mice that looked at changes between naïve females and mothers in the cortical entrainment to sequences of identical pup calls [27]. That study only analyzed total spike counts and found that auditory cortical MUs in mothers but not naïve females could follow sequences of pup calls up to the naturally occurring pup call repetition rate of ~5 Hz. It explored neither the information encoding of single pup calls nor changes in the timing of spiking information within the response to each call. Moreover, while better entrainment could arise from changes in the duration of the long after-hyperpolarization potential following a spiking response, the mechanisms responsible for the fine time-scale changes in spiking during the call (such as frequency sensitivity) are probably different. Nevertheless, both these works utilized natural control groups (virgins) to explore the impact that behavioral significance has on the neural code for vocalizations. This natural paradigm has not been exploited before, perhaps because a progression in the significance of an intraspecies vocalization is difficult to trace through the life of an animal.

An alternative approach to this would be to instrumentally train animals in specific behavioral tasks using unfamiliar vocalizations, such as from another species [13]. However, that may or may not generate the same type of plasticity as the natural context. On the one hand, training monkeys in a tactile discrimination task produced an earlier and larger pooled MU PSTH response for primary somatosensory cortical neurons after stimulation of the trained digit (behaviorally relevant) compared to an untrained digit (not behaviorally relevant) [37]. This is reminiscent of the differences between naïve females and mothers in the PSTH response auditory cortical MUs to a typical pup call (Figure 1B).

On the other hand though, there are reasonable arguments for why the plasticity may be different. First, training contexts usually familiarize an animal to only a small number of vocalization tokens, while communication sound learning likely involves a huge variety of exemplars from which statistical regularities are extracted [38]. Second, the reinforcement mechanisms in instrumental versus natural contexts could differ, depending on the nature and value of the reward. Supporting this, in the natural maternal context, young pups have been found to be more rewarding to a recent rat mother than cocaine [39]. Moreover, suckling pups stimulate a mother's dopaminergic reward system differently than cocaine does [40]. Hence, rewards derived from a social environment might produce different brain changes than food or water. In conjunction with this, an animal's hormonal state likely differs in natural and training contexts. This is relevant in light of a recent study showing that estrogen can modulate the auditory processing of behaviorally relevant song signals in the bird [41].

Indeed, training tasks have not always yielded the same kind of coding plasticity that is reportedly achieved naturally in a communication setting. For example, some marmoset auditory cortical neurons have a firing rate preference for the normal forward direction of a marmoset twitter call compared to its reverse [5], but water-restricted ferrets trained to recognize those same marmoset tokens in a go/no-go task do not [13]. Interestingly though, the temporal response patterns from the cortical neurons of trained ferrets do carry more information for classifying real and reversed call tokens compared to untrained animals [13]. One interpretation of these findings is that the specific plastic changes that are induced depend on the behavioral task [42]. In communication, multiple tasks, such as detection, discrimination, and categorization, are sometimes simultaneously necessary. This might require a different encoding of the vocalizations than what results from training animals in a specific task on specific exemplars with a specific form of reward. This reinforces the need to use new methods, such as the one implemented here, to evaluate the contributions that neurons make towards useful communication processing tasks.

Our conclusions were based on an information theoretic analysis of the responses to multiple vocalizations from within a single intraspecies communication sound category. Information theoretic analyses have been used previously to study the auditory cortical coding of sound location [43], generic sound [44,45], and animal vocalization [12,13] classification. Our focus though was on how natural acoustic variation is incorporated into communication processing. We considered two complementary psychophysical functions that must deal in different ways with this variability. Detection requires that neural responses be the same for different variations within the call category, while discrimination is best when they are reliably different. This dual approach goes beyond computing information just between responses and individual sounds, as has been done for vocalization ensembles with altered (e.g., time expanded or reversed) calls [12,13]. Instead, to quantify the detection and discrimination of a communication category, different real calls that sample the category's known acoustic variability should be presented.

Methodologically, we should point out that while calls were selected based on the distribution of acoustic parameters within our large library [19], we do not know the “true” likelihood that a given animal actually encountered each type of call or how rare calls are in the natural setting. Changes in these probabilities would affect the a priori entropy for discrimination and detection, respectively. However, since the same probabilities were assumed for all animals, and our conclusions were based on comparisons between animal groups, we did not feel this was a serious limitation.

Within such a paradigm, we found that sites could be better at conveying one type of information compared to another (off-diagonal points in the upper right quadrant of Figure 8). For example, onset responders tended to be better at detection than discrimination (for example, MU 528 and SU A). It will be interesting to see in future experiments whether neurons carrying different information might be spatially clustered in the auditory cortex, perhaps forming functional modules. In support of this possibility, a recent guinea pig study found similarities within an auditory cortical column in the response to an intraspecies call and segregation of different response types across the cortex [9].

The cortical encoding changes we found are only a first step in fully characterizing the differences that are correlated with the communicative significance of pup calls. For example, if there is significant synergy or redundancy in the neural responses, the information we computed in independent 2-ms bins would not predict how the full time course of the response might detect or discriminate the pup calls. This requires many more trials in order to accurately estimate the Shannon information when more response possibilities can occur (i.e., more spikes in larger bins). An alternative approach has been to classify neural responses according to a specific decoding algorithm and then to calculate information between the actual and assigned stimuli [13,35,46,47]. By the data-processing inequality [48], this would produce a lower bound on the true information between stimulus and response. Although we do not assume a decoder, our study is comparable in that it is also limited by the data-processing inequality due to the 2-ms binning. Nevertheless, even if the full detection or discrimination information is similar between mothers and naïve females, our results still suggest that changes occur in how this information is distributed across time.

Finally, it is important to note that mothers and naïve females show behavioral differences in the recognition of pup calls, as inferred from their relative preferences to approach these sounds in two-alternative choice tests [25,26]. It is not known though whether the “perceptual qualities” of the calls differ for the two animal groups. In fact, a preferred approach could in principle result from a change in the motivation or decision to respond to a stimulus, without any sensory changes. Yet our findings demonstrate that the sensory cortical neural encoding of communication sounds can not only change, but actually might enhance the neural ability to detect and discriminate calls once they are preferred. In this particular system, such plasticity might be behaviorally advantageous for retrieval performance in natural settings. In general, sensory improvements that increase the signal over the background noise may be an important preprocessing step for decisions to act.

## Materials and Methods

### Animal surgeries.

MU experiments on six recent mothers and six pup-naïve female CBA/CaJ mice (11–18 wk) were carried out at the University of California at San Francisco (UCSF); additional SU studies in two mothers and one pup-naïve female were conducted at Emory University. The Institutional Animal Care and Use Committees of both UCSF and Emory approved all procedures. Animals were housed under a reversed light cycle. Details of the surgery and setup have been described elsewhere [27,49]. Briefly, mice were anesthetized with a combination of ketamine (100 mg/kg initial dose and 65 mg/kg maintenance) and medetomidine (0.3 mg/kg) and secured in a nose clamp for a craniotomy over the left auditory cortex [50] and recording.

After surgery, animals were repositioned in front of a wide-bandwidth ribbon tweeter (High Energy EMIT-B, Infinity, http://www.infinitysystems.com) or a Tucker Davis Technology's (TDT, http://www.tdt.com) ES1 electrostatic speaker (Emory) in an anechoic chamber (Industrial Acoustics, http://www.industrialacoustics.com). The sound delivery system was calibrated by TDT software using a Brüel and Kjær (B&K, http://www.bksv.com) free-field microphone coupled to a B&K 2669 preamp and 2690 amplifier.

### Acoustic stimulus.

Stimuli were generated using TDT System 3 hardware and software (sample rate of 195,312.5 samples per second via an RP2.1 digital-signal processing module at UCSF and 223,214.2857 samples per second via an RX6 module at Emory) and presented through Brainware (http://www.brainware.com), which also served to collect thresholded action potentials. Noise bursts and frequency sweeps were used as search sounds to locate auditory responses. We used 60-ms tone pips of varying amplitude and frequency to estimate the CF and threshold for each recording site (details available in [27]).

Recordings of pup calls were drawn from a large library of natural ultrasound vocalizations from CBA/CaJ mice [19]. Recording snippets were high-pass filtered in software (25 kHz corner, eight-order Butterworth filter, MATLAB), spectrally denoised [19], multiplied by a 0.5-ms cos^2^ onset and offset function, and scaled to a target root-mean-square (RMS) amplitude to generate clean vocalizations for playback. Frequency-divided pup calls were generated in the same manner, except that a Hilbert transform was applied to extract the real call's phase function. This was multiplied by 0.5 before inverse transforming the signal with the original amplitude envelope to generate a frequency-halved call.

For MU recordings, unless otherwise noted, twelve trials (600 ms long) of each of the calls were presented in random order at all sites; during SU recordings the number of trials varied depending on how long units were held (see Figure 4 for details). Recordings of adult CBA/CaJ calls and synthetic narrowband noise models of the typical pup call (Figure 1) were also played back but were not analyzed in this work.

### Electrophysiology.

The exposed cortical area was photographed to record penetration locations. Epoxylite-coated tungsten microelectrodes (Fred Haer and Company, http://www.fh-co.com) were introduced perpendicularly into the cortex and advanced 300–600 μm below the surface. For MU recordings, electrode impedances were typically 1–2 MΩ; 4–10 MΩ electrodes were used in SU experiments.

Initial penetrations were directed towards the expected center of the auditory cortex. Subsequent penetrations (usually along the rostral-caudal axis) tried to locate the border between the primary (A1) and anterior auditory fields (AAF) by searching for a reversal of the tonotopic gradient. Once identified, we next tried to target the ultrasound field (UF) or the secondary auditory field (A2) and dorsal posterior (DP) fields, based on relative topography and response properties [27,50].

If neurons responded to tone frequencies above 20 kHz (which happened most of the time), we presented the pup call stimuli (at a few sites, only the typical pup call or the full pup call ensemble was played back, but not both). At a random subset of sites, frequency-halved pup calls were also presented. Some sites did not have a readily identifiable tuning curve, although they were driven by sounds.

In total, MU responses to either the typical pup call (Figure 1) or the pup call and frequency-divided pup call ensembles were collected from 112 sites in mothers and 106 sites in naïve females. The target RMS amplitude for the typical call was 65 dB sound pressure level (dBSPL). For the call ensembles, the target was 74 dBSPL at most sites and 65 dBSPL at a few others. The inclusion of the 65 dBSPL data did not affect our conclusions, so data from both sound levels were combined.

Since complete auditory cortical maps were not obtained, we were not always able to unambiguously determine the likely auditory field for each site. Our population in mothers (naïve females) included 54 (32) primary (i.e., A1 or AAF), 18 (12) UF, 5 (4) A2, and 35 (58) other/uncertain sites (classification as in [27]).

### Information analysis.

Although Figures 5 and 6 illustrate detection and discrimination information in terms of the reduction in stimulus uncertainty, information was estimated in practice by applying Bayes' Theorem [48] and looking at response uncertainty. Thus, the Shannon information was defined as the difference between the response entropy *H*(*r*) = *p*(*r*)*log*_2_ *p*(*r*) and the response entropy conditioned on the stimulus *H*(*r|s*) = *p*(*r|s*)*log*_2_ *p*(*r|s*), where *p*(*r*) is the probability of response *r* (zero, one, two, . . . spikes in a 2-ms bin), and *p*(*r|s*) is the probability of response given the stimulus *s*.

These quantities were estimated through data-size scaling procedures, wherein trials were considered first together and then randomly partitioned into two, three, and four groups [28]. For each data size (1, 1/2, 1/3, and 1/4), the entropies were calculated (from the probabilities as described below) and averaged together. These were then fit to a quadratic function of the inverse data size to extract an infinite data limit [51]. The difference between the fitted response and conditional entropies provided one estimate of the information. The procedure was then repeated 50× with different random data partitions, and the final information estimate averaged these. Although our methods can in principle be extended to bin sizes larger than 2 ms, this resolution was used to limit the possible responses so that probabilities could be more accurately estimated from the relatively small number of trials.

For detection, *p*(*r|s* = *“call”*) at time bin *t* was found by grouping all trials for all pup calls together (usually 12 trials × 18 calls = 216 effective trials), since the identity of individual calls was ignored. The probability for a specific spike count (e.g., one spike) in that bin was defined as the number of trials having that spike count, divided by the total number of effective trials. A time-invariant *p*(*r|s* = *“no call”*) was estimated by drawing the same number (e.g., 216) of response time bins from random times in the spontaneous activity. The randomized detection information at time bin *t* was computed by randomly assigning the call and spontaneous trials to either the “call” or “no-call” stimulus. For discrimination, the probabilities were estimated from the trials to each individual call. The randomized discrimination information was computed by randomly assigning those trials to the different calls. For frequency (duration) information, the six calls lying within the same frequency (duration) range were grouped together, forming three different frequency (duration) ranges.

It should be pointed out that when probability distributions must be inferred from finite trials, all information estimates are subject to bias [52]. Although techniques such as data-size scaling have been developed to minimize it, non-negligible biases can be present when there are large numbers of probable responses (those with nonzero likelihood) or a large number of stimuli, as in the case of call discrimination. This is probably why the examples in Figure 7L–7V show an offset in the time course of discrimination information long after the stimulus turned off (for both the actual and randomized control information). Importantly, the biases implied by these late-period offsets were not necessarily the same as the biases at the time of the peak information, since bias is sensitive to the exact probability distribution of the responses.

To convince ourselves that our conclusions based on the peak information values were not affected by this, we tested several different bias-correction methods. In addition to the data-size scaling procedure described above, we also checked our results using a data-size scaling procedure with (1, 11/12, . . . , 6/12) random partitions of the data (again averaging together the estimates from 50 different randomizations). We also tried the so-called naïve-bias correction procedure described in [31]. By performing simulations that assumed the empirical response probability distribution from one of our recording sites was the true probability distribution, we found that the two data-size scaling procedures reasonably estimated the true discrimination information when this was above ~0.1 bits (errors up to ~0.02 bits for true information near 0.1 bits and improving to <0.003 bits for progressively larger true information). The naïve-bias correction methods suffered greater systematic bias. We also applied the best-upper-bound (BUB) information estimate developed by Paninski [52] to our data but found this to yield generally much larger biases. Hence, we relied on the data-size scaling method and chose a threshold of 0.1 bits for the discrimination situation (bias was not a concern in the other situations), above which we considered our information estimate to be valid. While higher thresholds could be chosen, we decided against this since it left too few sites in the naïve female group to yield statistically sound comparisons.

It is important to note that even when the alternative bias-correction methods were applied to all our data, our conclusions were still the same. This is probably because our analysis was dependent not so much on the absolute value of the information but rather on the relative information values compared across the two animal groups. The differences between the two groups were apparently sufficiently large to emerge regardless of how we attempted to correct the bias.

### MU analysis.

Our population comparisons were based on the MU recordings. To test whether our main conclusions would be sensitive to this, we looked at five sites in both mothers and naïve females that had the largest peak information (considering detection and discrimination separately). Since the threshold level for detecting action potentials could affect how many units contributed to a recording, we systematically increased that level to reduce the overall spike counts for a site to approximately 50%, 25%, and 10% of the original recording. Thus, at the 10% level, we should only have been including the largest spikes at a site. When information was recalculated, the peak times in nearly all cases stayed the same, suggesting that the difference in timing observed between mothers and naïve females was not very sensitive to the MU nature of the data. In fact, the peak information times for SU A and SU B in Figures 9 and 10 agreed well with the MU distribution for mothers.

## Abbreviations

CF: characteristic frequency
dBSPL: dB sound pressure level
KS: Kolmogorov-Smirnov
MU: multiunit
n.s.: not significant
PSTH: peristimulus time histogram
SU: single unit
